# Supplementary material for: HIV and Schistosoma Co-Exposure Leads to Exacerbated Pulmonary Endothelial Remodeling and Dysfunction Associated with Altered Cytokine Landscape
Source: Cells. 2022 Aug 4;11(15):2414. doi: 10.3390/cells11152414 (PMC9368261; doi:10.3390/cells11152414)
Supplement: Supplementary file 1 [file cells-11-02414-s001.zip › cells-1755501-supplementary.pdf]

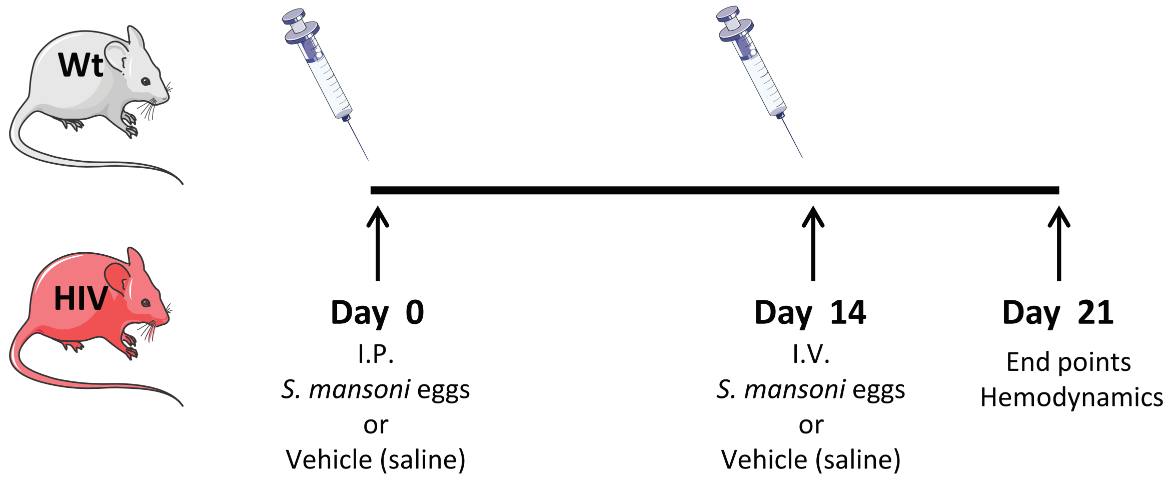

Supplementary Figure S1. Outline of administration of *S. mansoni* eggs and mouse analysis.

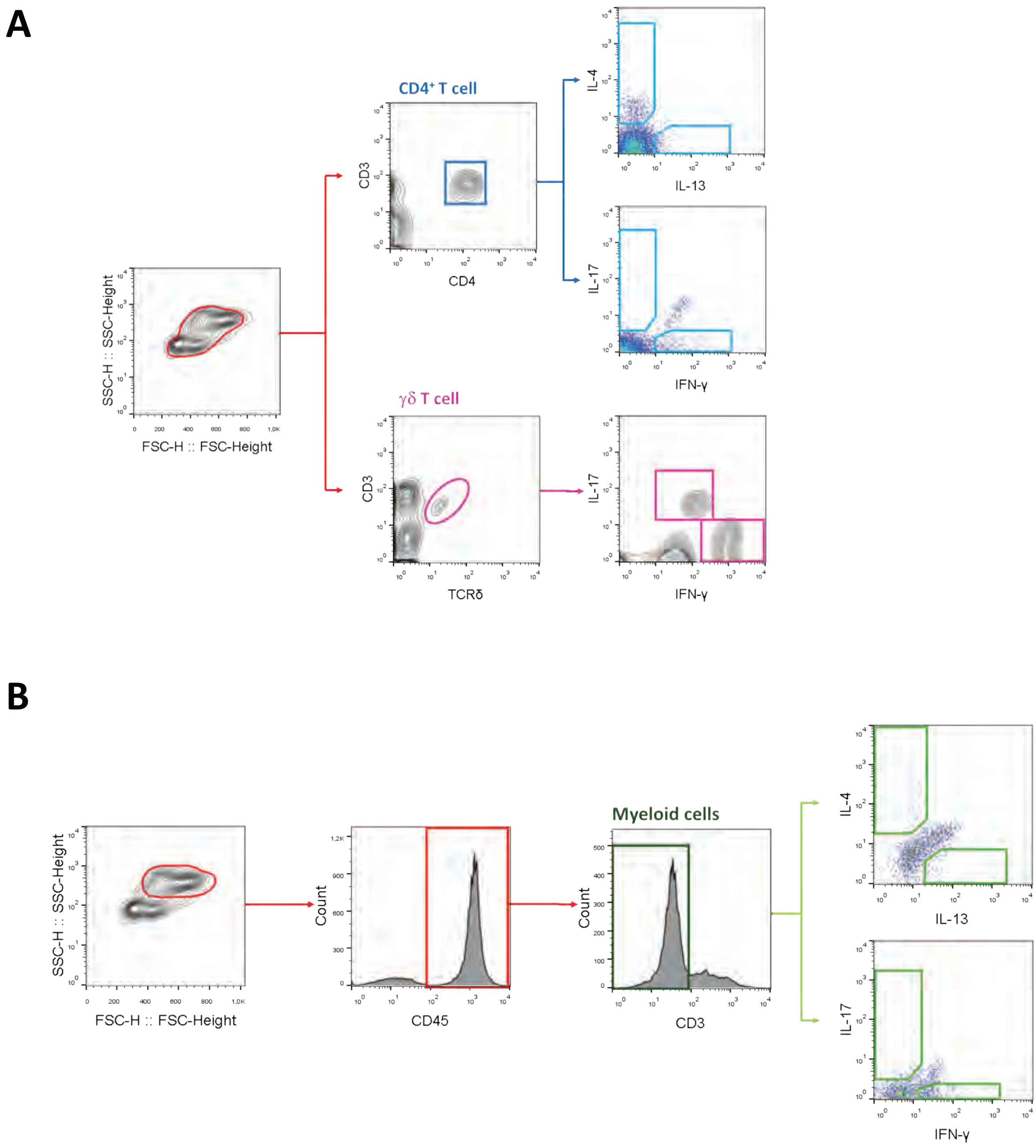

Supplementary Figure S2. Flow cytometry gating strategy for identification of cytokine-expressing (A) T cells and (B) myeloid cells in the lung.

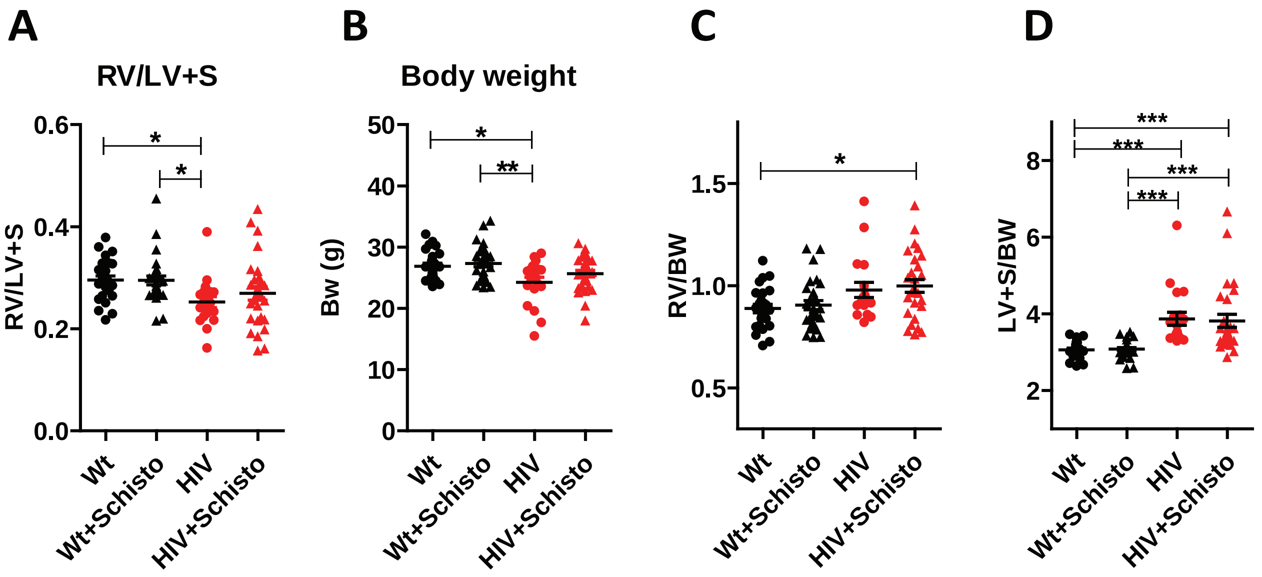

Supplementary Figure S3. Assessment of cardiac remodeling in Wt and HIV mice following exposure to schistosome eggs. (A) and (C): right ventricular (RV) weight relative to left ventricle + septum (LV + S) or to body weight (BW), respectively. (B) Body weight and (D) left ventricular and septum weight relative to body weight (BW) in Wt and HIV mice unexposed or exposed to schistosome eggs. Results are means ± SEM (n=19-28). *$P<0.05$, **$P<0.01$ and ***$P<0.001$ (one-way ANOVA analysis followed by Tukey post hoc test).

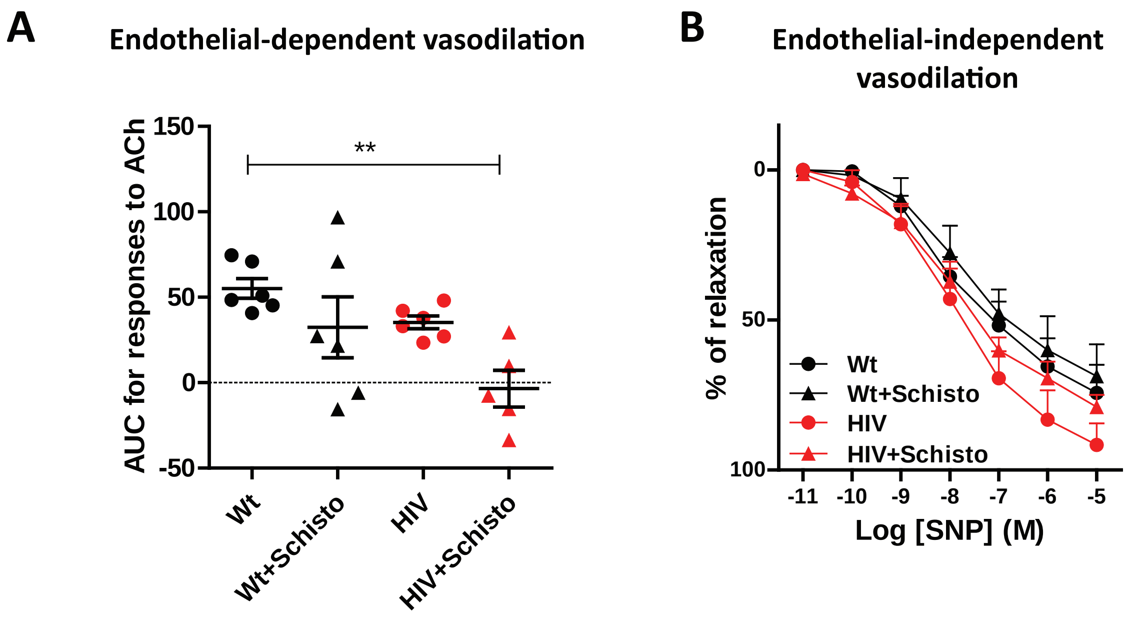

Supplementary Figure S4: Endothelial dysfunction in HIV mice is aggravated by Schistosoma egg exposure. Panel (A) shows the mean values of the area under the curve (AUC) of ACh-induced relaxa-tion. The dotted line represents zero relaxant response to ACh. (B) Relaxant effects of the endothelium independent vasodilator sodium nitroprusside (SNP) in serotonin-stimulated PA. Values are mean ± SEM (n=5-6). **P<0.01 as determined one-way ANOVA analysis followed by Tukey post hoc test (panel A).

A

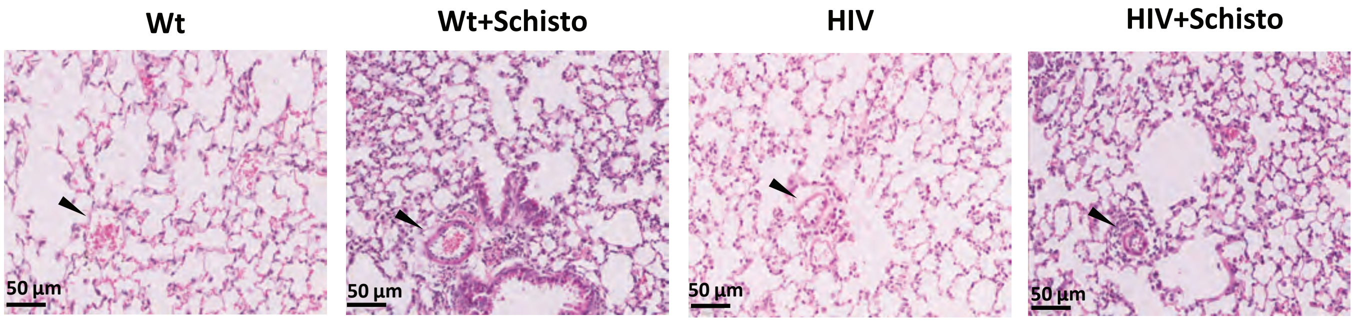

Supplementary Figure S5. Representative hematoxylin-eosin staining of paraffin-embedded lung sections from Wt and HIV mice exposed or unexposed to Schistosoma (Solid arrowheads mark pulmonary arteries).

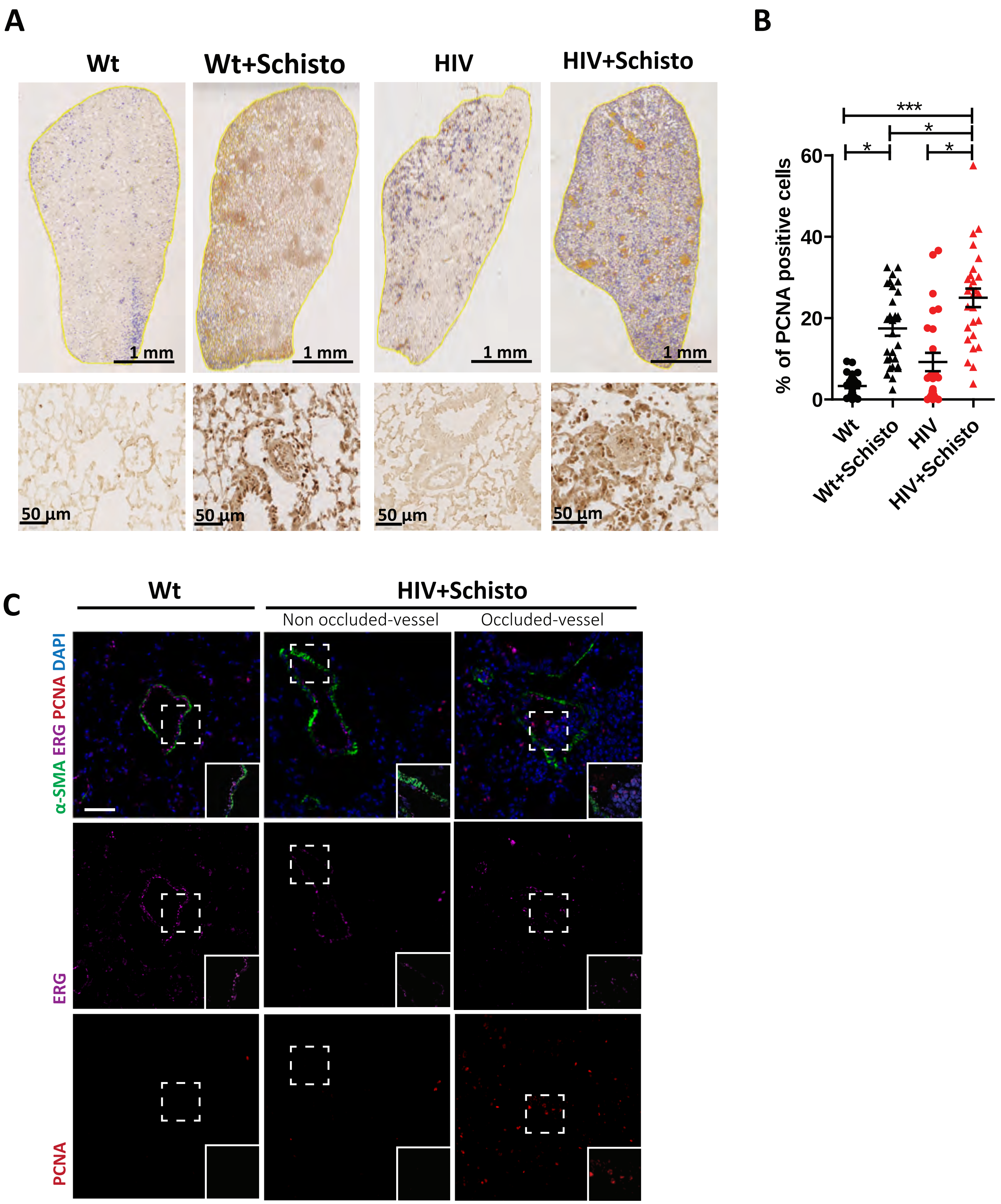

Supplementary Figure S6. Co-exposure to Schistosoma and HIV enhance cellular proliferation in the lungs. (A) Representative images of proliferating cell nuclear antigen (PCNA) in lung sections and amplified vessels from Wt and HIV mice exposed and unexposed to Schistosoma eggs. (B) Quantification of the PCNA positive cells within the vessels area (n=25-28 from 5 mice in each group). (C) Representative immunofluorescence images of α-SMA (green), ETS Transcription Factor (ERG) (violet) and PCNA (red) staining in lungs of Wt and HIV-Schisto in non-occluded and occluded vessels, including higher magnifications (white squares). Scale bar 50 μm. Results are expressed as mean ± SEM. *P<0.05 and ***P<0.001 (one-way ANOVA analysis followed by Tukey post hoc test).

A

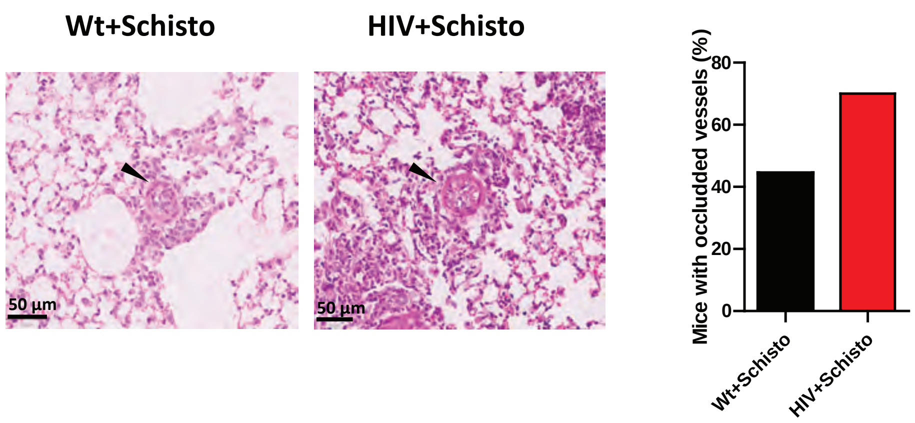

Supplementary Figure S7: Increased vessel obliteration and plexiform-like lesions in HIV mice exposed to parasite eggs. (A) Representative photomicrographs of hematoxylin and eosin stained occluded vessels (Solid arrowheads) (left panels). Frequencies of animals with occluded vessels (middle panel) and percentage of occluded vessels (right panel) (n= 19 mice per group).
